# A Bayesian framework systematic review and meta-analysis of anesthetic agents effectiveness/tolerability profile in electroconvulsive therapy for major depression

**DOI:** 10.1038/srep19847

**Published:** 2016-01-25

**Authors:** Guillaume Fond, Djamila Bennabi, Emmanuel Haffen, Lore Brunel, Jean-Arthur Micoulaud-Franchi, Anderson Loundou, Christophe Lançon, Pierre-Michel Llorca, Pascal Auquier, Laurent Boyer

**Affiliations:** 1 Université Paris Est-Créteil, Pôle de psychiatrie des hôpitaux universitaires H Mondor DHU Pe-PSY, INSERM U955, Eq Psychiatrie translationelle, Fondation FondaMental Fondation de coopération scientifique en santé mentale, Créteil, France; 2 Service d’explorations fonctionnelles du système nerveux, Clinique du sommeil, CHU de Bordeaux, Place Amélie Raba-Léon, 33076 Bordeaux, France; 3 Aix-Marseille University, EA 3279 Research Unit - Public Health: Chronic diseases and quality of life, Marseille, France; 4 Department of Epidemiology, Timone University Hospital, APHM, Marseille, France; 5 Department of Clinical Psychiatry, CIC-1431, University Hospital of Besançon, EA Neurosciences 481, University of Franche-Comté, Besançon, France; 6 Network of Expert centres, FondaMental Foundation, Créteil 94000, France; 7 University Hospital of Clermont-Ferrand, Clermont-Ferrand, France

## Abstract

The aim of this study was to assess the efficacy and tolerability/acceptability of 6 anesthetic agents in ECT for depressive disorders. We systematically reviewed 14 double-blind randomized controlled trials (610 participants). Efficacy was measured by the mean scores on validated depression scales at 6 ECT (or the nearest score if not available), number of responders at the end of treatment and seizure duration. The acceptability was measured by the proportion of patients who dropped out of the allocated treatment, and the tolerability by the number of serious adverse events and post-treatment cognition assessment. After excluding the trials responsible for heterogeneity, depression scores of patients who were administered methohexital were found to be significantly more improved than those who received propofol (p = 0.001). On the contrary, those who were administered propofol had lower depression scores than those with thiopental at the end of treatment (p = 0.002). Compared to propofol, methohexital was found to be significantly associated with higher seizure duration (p = 0.018). No difference was found for the acceptability profile (all p > 0.05). In summary, ketamine and methohexital may be preferred to propofol or thiopental in regard of effectiveness in depression scores and increased seizure duration. Further studies are warranted to compare ketamine and methohexital.

Major depressive episode is a common mental disorder. It affects millions of people worldwide and is considered by the World Health Organization (WHO) to be one of the leading causes of disability^1^. Electroconvulsive therapy (ECT) is a well-established treatment for severe depressive episode^2^,^3^,^4^. Intravenous anesthetic medication before ECT is used to minimize subjective unpleasantness and adverse side effects of the induced tonic-clonic seizure. Almost all anesthetic agents have anticonvulsant properties because of their effects on the gamma-aminobutyric acid receptors and may, therefore, influence seizure variables and clinical outcome of ECT^5^. The choice of anesthetic agent usually depends on seizure duration, hemodynamic, and recovery parameters. Anesthesia with propofol, one of the most currently used anesthetic agents in ECT, was suggested to have a significant reducing effect on seizure duration during the course of ECT, which may result in more inadequate seizures, despite the use of a higher mean stimulus charge^6^.

Beyond seizure duration, the influence of anesthetic medications on the successful reduction of depressive symptoms and adverse events is unclear. Ketamine was recently reported to have specific short-term antidepressive properties in both ECT and non-ECT studies^7^,^8^. However, it remains rarely used for anesthesia in clinical practice, due to potential side-effects, namely severe blood pressure increase, manic switches in bipolar patients, confusion and prolonged delirium^9^,^10^,^11^,^12^.

The objective of the present systematic review and multiple-treatment meta-analysis, which accounts for both direct and indirect comparisons, was to assess effectiveness, acceptability and tolerability of 6 anesthetic agents used in ECT for major depression.

## Methods

This protocol was registered in the PROSPERO registry under the registration number CRD42015016428.

### Study selection and data collection

For our analysis, we included only randomized controlled trials (RCTs) comparing any of the following 6 anesthetic agents for induction of pre-ECT anesthesia: etomidate (ETO), ketamine (KET), methohexital (METH), midazolam (MID), propofol (PROP), thiopental (THIO). To identify the relevant studies, we reviewed the following databases: PubMed (from 1966), Embase (from 1980), PsychINFO (from 1806), BIOSIS (from 1926), Science Direct (from 2006), Cochrane CENTRAL (from 1993), Cochrane collaboration depression, anxiety, and neurosis review group controlled trials registers (CCDANDTR studies and CCDANCTR-references) up to Jan 30, 2015.

We asked study investigators to supply all available information. Within the reviewing team, GF and DB independently reviewed references and abstracts retrieved by the search, assessed the completeness of data abstraction, and confirmed quality rating. We used a structured data abstraction form to ensure consistency of appraisal for each study. Investigators were contacted and asked to provide data to supplement the original articles.

### Criteria for selecting articles

Studies were included if they met the following criteria:

- Design: randomized double blind controlled trials;
- Intervention: anesthetic administration (one administration or more, alone or with other anesthetic agent) followed by one ECT session;
- Participants: participants with a diagnosis of depression (major depressive disorder MDD or bipolar depression BD, resistant or not) according to an international classification (Diagnostic and Statistical Manual DSM or International Classification of Diseases ICD);
- Evaluation of depression severity based on a validated scale. All validated scales assessing depression were included (Hamilton depression rating scale (HDRS)^13^, Montgomery-Asberg depression rating scale (MADRS)^14^ or Beck Depression Inventory (BDI)^15^). When trials reported results from multiple rating scales, HDRS scores were chosen.

There was no language or date restriction. The manuscripts with the following criteria were excluded: (i) absence of comparison between groups with two different anesthetic agents administration; (ii) the study enrolled subjects with “narrow” diagnoses (e.g., postpartum depression) or secondary depression (e.g., vascular depression) (iii) the study did not report raw data or the authors did not provide raw data. When eligible studies were missing key data, their corresponding authors were contacted at least twice by e-mail at 2-week intervals.

### Outcome measures

#### Primary outcomes

Efficacy was proxy by the difference between baseline (before first ECT) and the 6^th^ ECT on validated depression scales. When the score after 6 ECT was not available, the nearest score was chosen. The number of ECT was taken into account in the qualitative interpretation of the results.

Efficacy was also proxy by responder rates at the end of the study (we gave preference to the timepoint given in the original study as the study endpoint). The definition of responder was taken into account, as well as the mean number of ECT in each group.

Seizure duration was included as an efficacy outcome, as seizure duration may play an important role in ECT effectiveness^16^.

Acceptability was proxy by the rate of patients who terminated the study early for any reason during the study (dropout rate).

Tolerability was proxy by number of adverse events and cognitive outcomes assessed by validated tests after ECT treatment. All tests assessing at least one cognitive function were included.

### Selection of studies and data extraction

Two authors (G.F and D.B) screened titles and abstracts of database records and retrieved full texts for eligibility assessment and independently checked the full text records for eligibility. Disagreements were resolved by consensus discussion.

The manuscripts of the studies were then independently reviewed by two of the authors (G.F. and D.B.). Data was independently extracted into a standard electronic form: first author name, date of publication, country, sample size, depression assessment scales, definition of responder, number of patients with major depression and bipolar depression, diagnoses, ECT lead treatment, number of ECT treatments before depression post-treatment evaluation, anesthetic treatment and dose of each arms. Any discrepancies were resolved by consensus with a third reviewer (L.B).

### Assessing the methodological quality of included studies

The methodological quality of included studies was assessed independently by two of the authors (G.F. and D.B.). Any discrepancies were resolved by consensus with a third reviewer (L.Boyer).

First, we used markers of internal validity from the Cochrane Risk of Bias Tool^17^. The risk of selection bias was assessed at study level (sequence generation, allocation sequence concealment), the risk of performance bias at comparison level (blinding of medical personnel) and the risk of detection bias as well as attrition bias were assessed at outcome level (blinding of outcome assessors, handling incomplete outcome data). Studies’ risk of bias could then be qualified as low, unclear or high.

Second, we further classified studies according to their level of evidence using the classification scheme requirements for therapeutic questions (Gross and Johnston 2009). The level of evidence was classified using a four-classes system (Class I through Class IV), with Class I indicating the strongest evidence and Class IV the weakest.

### Statistical Analyses

Depression scores decreases and seizure duration were analyzed using the standardized mean difference (SMD) with 95% confidence intervals (CIs) for each study. SMD were defined as the difference in mean outcome between two groups divided by the pooled standard deviation of the measurements. Responder and dropout rates were analyzed using the odd ratio (OR), defined as the ratio of the odds of the studies event (i.e., responder or dropout) between two groups. Given the diversity of drugs, administered doses and cognitive tests, adverse events and cognitive function outcomes were qualitatively described.

First, we did head-to-head direct evidence, also called par-wise meta-analyses. We synthetized studies that compared identical interventions (i.e., KET vs. THIO, KET vs. PROP, PROP vs. METH, and PROP vs. THIO) using a random effect model^18^. This models accounts for between-study heterogeneity by weighting studies similarly. Heterogeneity was assessed using the I2 statistic, which represents the percentage of variance due to between-study factors rather than sampling error^19^. We considered values of I2 >50% as indicative of large heterogeneity^20^. When possible, we used funnel plots and the Egger regression intercept (i.e., which assesses the degree of funnel plot asymmetry by the intercept from regression of standard normal deviates against precision) to estimate risk of bias^21^. Forest plots were generated to show SMD or OR with corresponding confidence intervals (CIs) for each study and the overall random effects pooled estimate. When possible, we performed sensitivity analyses according to the following variables: quality of the included studies, administered doses, ECT lead placements, inclusion of patients with bipolar depression. Analyses were performed with comprehensive meta-analysis software (version 2.0, National Institute of Health)^22^.

Second, we did network meta-analyses or mixed treatment comparisons for each outcome. Using a Bayesian hierarchical random-effect model with noninformative prior hypothesis, all direct and indirect comparisons were taken into account to reach a single consistent estimate of the effect of all included treatment based on all included studies. Consistency was assessed using the node-splitting method. A value near zero indicated that the comparisons in the network were consistent. Analyses were performed with the gemtc-R package and Winbugs^23^,^24^.

### Role of the funding source

None of the included studies was sponsored. No drug manufacturing company was involved in the study design, data collection, data analysis, data interpretation, writing of the report, or in the decision to submit the report for publication. All authors saw and approved the final version of the manuscript. The corresponding author had full access to all data and decided to submit for publication.

## Results

### Literature Search

**Figure 1:**
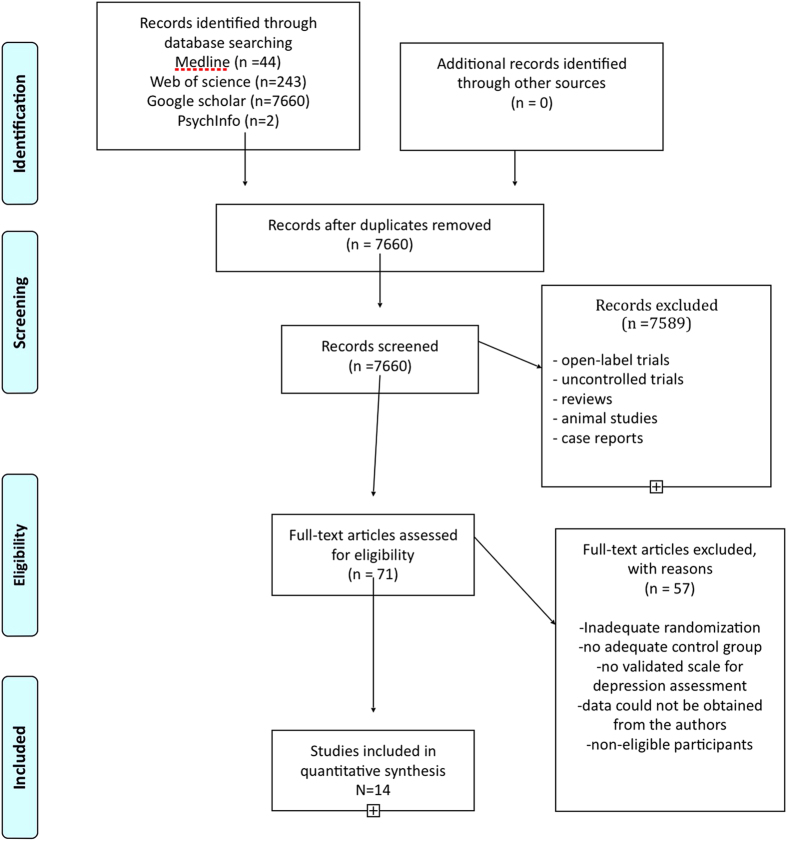
Prisma flow chart.

Our literature review is detailed in Fig. 1. Study quality was assessed using the Cochrane Collaboration’s Tool for Assessing Risk of Bias^17^ concomitantly to eligibility. We identified 71 studies, 14 of which met inclusion criteria for the present investigation^9^,^10^,^11^,^12^,^25^,^26^,^27^,^28^,^29^,^30^,^31^,^32^,^33^,^34^.

### Included RCTs: Main Characteristics

Fourteen RCTs were included in our quantitative meta-analysis, totaling 610 subjects with a major depressive episode (n = 545 with MDD; n = 65 with BD). Only one study included resistant depression in inclusion criteria^9^ (Tables 1 and 2).

One of the included studies was stopped for futility at a planned interim analysis^25^. The daily-administered doses ranked from 0.2 mg/kg (ETO), 0.4–2 mg/kg (KET), 1.05–1.43 mg/kg (METH), 1.32–1.72 mg/kg (PROP), 2–3.5 mg/kg (THIO). KET was the only anesthetic agent that was co-administered with other anesthetic agent in three RCTs (respectively THIO^10^,^25^ and PROP^9^).

Depression was assessed using Hamilton depression rating scale (HDRS)^13^ (8 studies), Montgomery-Asberg depression rating scale (MADRS)^14^ (4 studies) or Beck Depression Inventory (BDI)^15^ (2 studies). When trials reported results from multiple rating scales, we chose the HDRS scores, which were the most consistently reported estimates.

Thirteen out of the 14 RCTs were classified as level I or II (best quality) according to the classification scheme requirement for therapeutic questions. Only one study was classified as high risk of bias according to the Cochrane Risk of Bias tool, due to the lack of reported data and the impossibility to contact authors^34^.

### Efficacy: baseline and post-treatment depression scores

**Figure 2:**
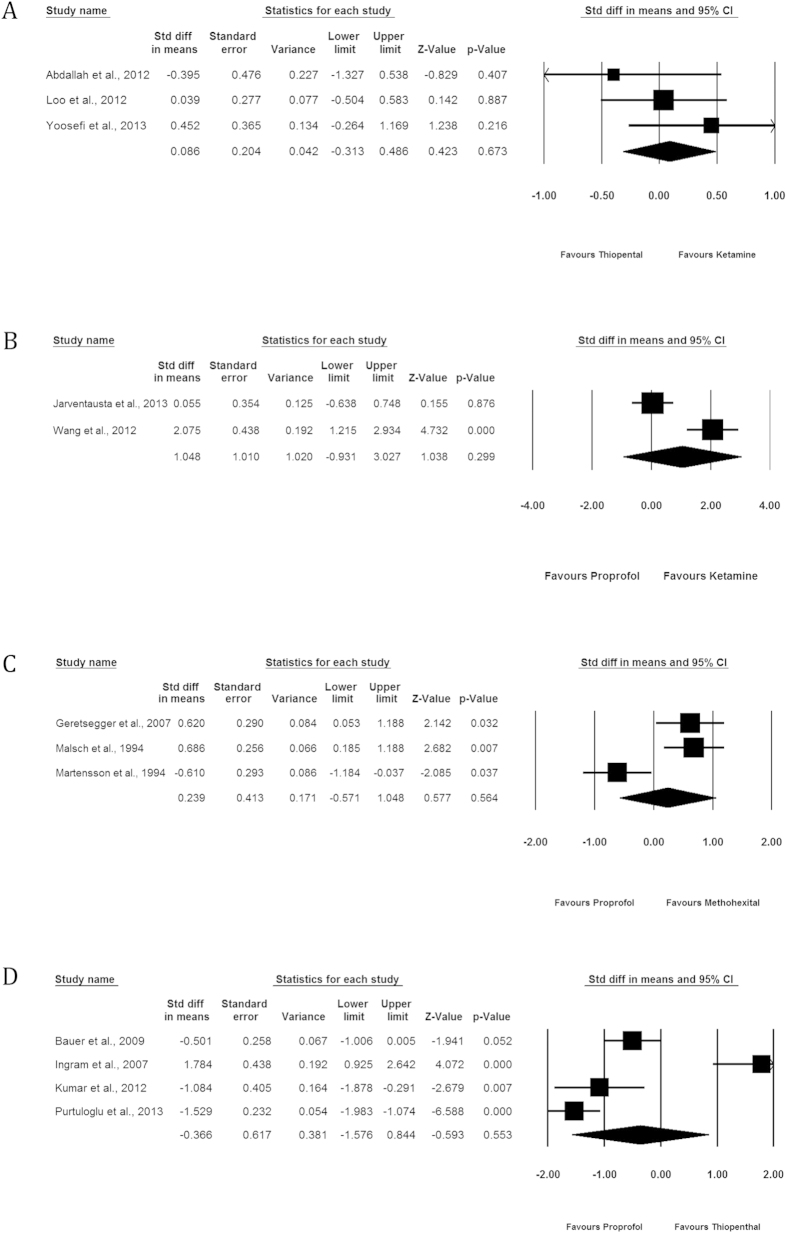
Comparative effectiveness of the 6 anesthetic agents (proxy by depression score improvement at 6 ECT or the nearest score if not available). (**A**) Ketamine vs Thiopental. (**B**) Ketamine vs Propofol. (**C**) Propofol vs Methohexital. (**D**) Propofol vs Thiopental.

The baseline and post-treatment depression scores were available for the 14 RCTs. (Fig. 2).

#### Ketamine vs Thiopental

We identified 3 studies comparing the effect of KET administration (n = 52) to THIO (n = 49) on depression scores^10^,^12^,^25^. In two of them, KET was co-administered with THIO in the KET group^10^,^25^. Overall, depression scores were significantly improved in each group, but there was no significant difference between the two groups (SMD = 0.086, 95% CI −0.313; 0.486, p = 0.673, I2 = 2.545%).

The associated funnel plot was reasonably symmetrical, although the limited number of studies does not allow the exclusion of publication bias (Appendix). The p value of the Egger’s regression intercept was >0.05.

#### Ketamine vs Propofol

We identified 2 studies comparing the effect of KET administration (n = 32) to PROP (n = 32) on depression scores^9^,^11^. There was no significant difference between the two groups (SMD = 1.048, 95% CI −0.931; 3.027, p = 0.299). In one study, 0.4 mg/kg S-KET was combined with THIO in the case group^9^, in the other KET 0.8 mg/kg was administered alone^11^.

The low number of studies (n = 2) does not allow to perform the analysis of the publication bias.

#### Propofol vs Methohexital

We identified 3 studies comparing the effect of METH administration (n = 86) to PROP (n = 78) on depression scores^29^,^32^,^33^. There was no significant difference between the two groups (SMD = 0.239, 95% CI −0.571; 1.048, p = 0.564, I2 = 84.791%).

The associated funnel plot was reasonably symmetrical, although the limited number of studies does not allow the exclusion of publication bias (Appendix). The p value of the Egger’s regression intercept was >0.05.

The heterogeneity was driven by one study^33^, that used MADRS as depression scale, contrary to the two others that used HDRS. When analyses were repeated after removing this study, a significant SMD of 0.657 (95% CI 0.282; 1.033, p = 0.001) was observed in favor of METH compared to PROP.

#### Propofol vs Thiopental

We identified 4 studies comparing the effect of PROP administration (n = 111) to THIO (n = 105) on depression scores^28^,^30^,^31^,^34^. There was no significance difference between the two groups (SMD = −0.366, 95% CI −1.576; 0.844, p = 0.553, I2 = 93.523%).

The associated funnel plot was rather assymmetrical, suggesting a possible publication bias (Appendix). However, the p value of the Egger’s regression intercept was >0.05.

The heterogeneity was driven by two studies^30^,^34^. One of these studies was classified as high risk of bias^34^ (Table 2), which may explain the heterogeneity for this study. The following discrepancies in methodology may explain the heterogeneity between the three remaining studies: the countries where studies were carried out, the proportion of patients with bipolar depression (Table 1), HDRS cut-off at inclusion (50% vs 60%), anesthetic dosage (variable vs. fixed dosages) (Table 3). When analyses were repeated after removing these two studies, a significant SMD of −0.669 (95% CI −1.096; −0.243, p = 0.002) was observed in favor of PROP compared to THIO.

### Efficacy: responder rates

**Figure 3:**
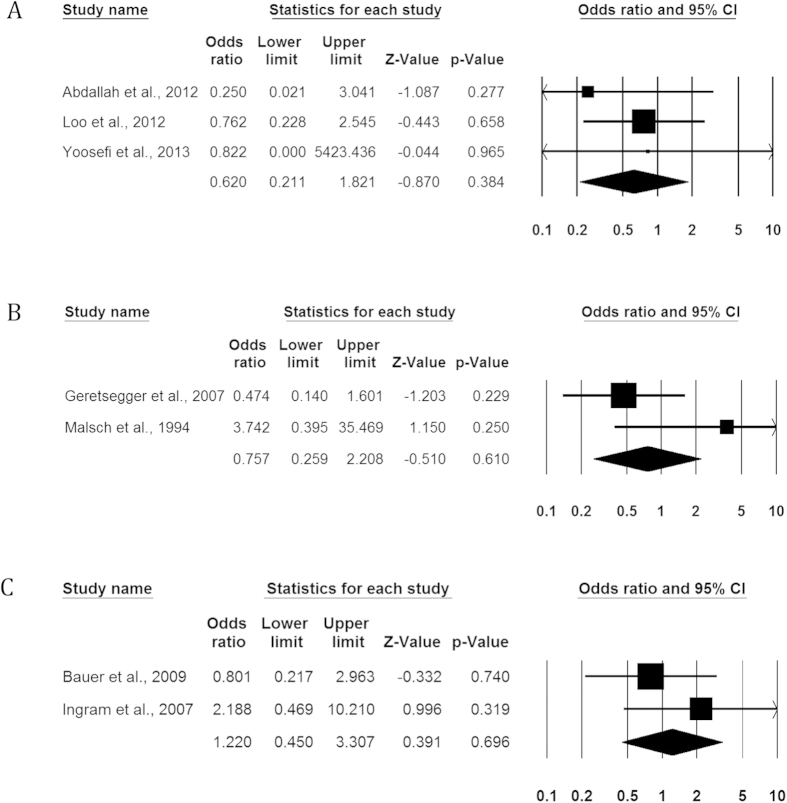
Comparative efficacy of anesthetic agents for ECT induction in major depression, proxy by response rates at the end of treatment. (**A**) Ketamine vs Thiopental. (**B**) Propofol vs Methohexital. (**C**) Propofol vs Thiopental.

The data for responder rates was available for 9 studies. (Table 3, Fig. 3).

#### Ketamine vs Thiopental

We identified 3 studies comparing the effect of KET administration (n = 52) to THIO (n = 49)^10^,^12^,^25^. There was no significant difference between the two groups (OR = 0.620, 95% CI 0.211; 1.821, p = 0.384, I2 = 0.00%).

The associated funnel plot was reasonably symmetrical, although the limited number of studies does not allow the exclusion of publication bias (Appendix). The p value of the Egger’s regression intercept was >0.05.

When analyses were repeated after removing the study of Yoosefi *et al.*^12^ (only MDD patients contrary to the two other studies which included MDD and BD), a non-significant OR of 0.617 (95% CI 0.208; 1.828, p = 0.384) was observed.

#### Propofol vs Methohexital

We identified 2 studies comparing the effect of METH administration (n = 60) to PROP (n = 55) on responder rate^29^,^32^. There was no significant difference between the two groups (OR = 0.757, 95% CI 0.259; 2.208, p = 0.610, I2 = 60.1%).

#### Propofol vs Thiopental

We identified 2 studies comparing the effect of PROP administration (n = 49) to THIO (n = 43) on responder rate^28^,^30^. There was no significant difference between the two groups (OR = 1.220, 95% CI 0.450; 3.307, p = 0.696, I2 = 0%).

### Efficacy: seizure duration

**Figure 4:**
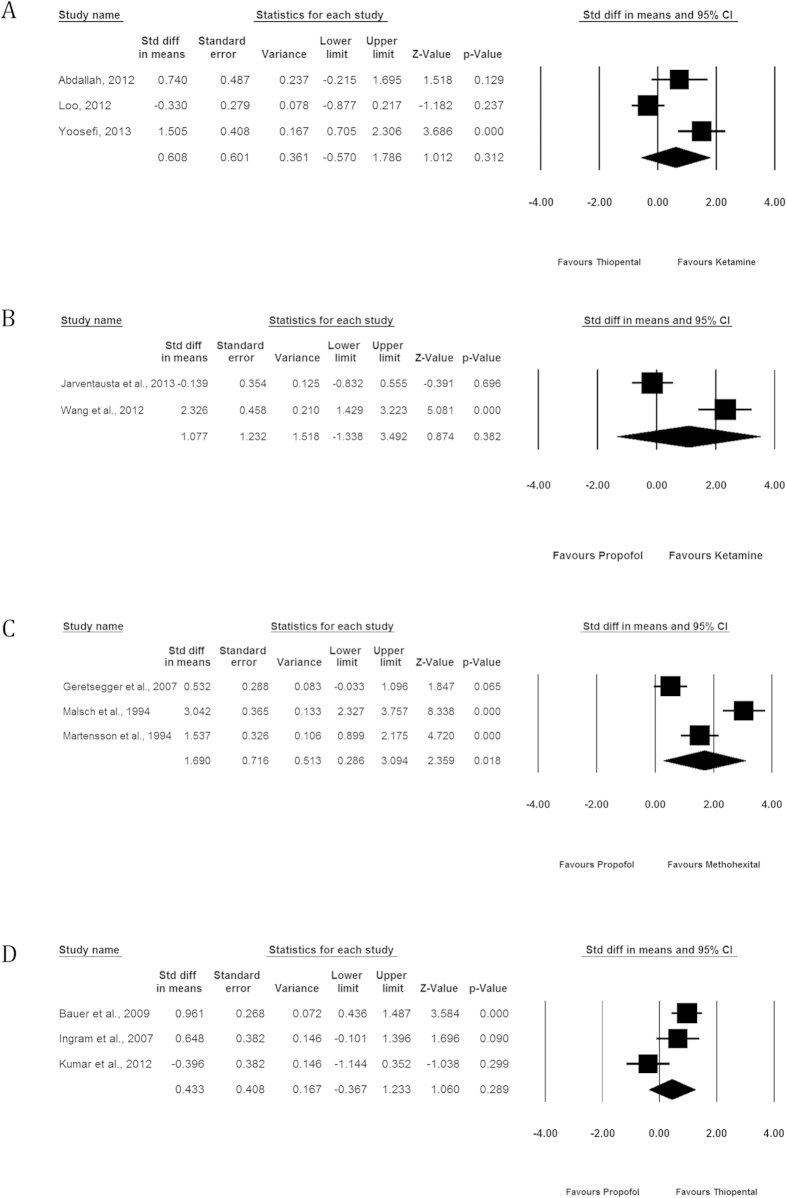
Comparative mean seizure duration associated with the administration of each anesthetic agent. (**A**) Ketamine vs Thiopental. (**B**) Ketamine vs Propofol. (**C**) Propofol vs Methohexital. (**D**) Propofol vs Thiopental.

The baseline and post-treatment depression scores were available for 13 RCTs. (Fig. 4).

#### Ketamine vs Thiopental

We identified 3 studies comparing the effect of KET administration (n = 52) to THIO (n = 49) on seizure duration^10^,^12^,^25^. There was no significant difference between the two groups (SMD = 0.608, 95% CI −0.570; 1.786, p = 0.312, I2 = 86.27%).

The associated funnel plot was reasonably symmetrical, although the limited number of studies does not allow the exclusion of publication bias (Appendix). The p value of the Egger’s regression intercept was >0.05.

After removing one study responsible for heterogeneity^10^, KET was associated with a longer seizure duration compared to THIO (SMD = 1.169, 95% CI 0.424; 1.914, p = 0.002; I2 = 31.04%).

After removing one study with high risk of bias^12^, no significant difference was found between groups (SMD = 0.130, −0.908; 1.169, p = 0.806; I2 = 72.45%).

#### Ketamine vs Propofol

We identified 2 studies comparing the effect of KET administration (n = 32) to PROP (n = 32) on seizure duration^9^,^11^. There was no significant difference between the two groups (SMD = 1.077, 95% CI −1.338; 3.492, p = 0.382, I2 = 94.49%).

#### Propofol vs Methohexital

We identified 3 studies comparing the effect of MET administration (n = 86) to PROP (n = 78) on seizure duration^29^,^32^,^33^. MET was found to be significantly associated with higher seizure duration compared to PROP (SMD = 1.690, 95% CI 0.286; 3.094, p = 0.018, I2 = 93.150%).

The associated funnel plot was reasonably symmetrical, although the limited number of studies does not allow the exclusion of publication bias (Appendix). The p value of the Egger’s regression intercept was >0.05.

This SMD remained significant after removing one high risk publication bias study^32^ (SMD = 1.023, 95% CI 0.038; 2.008, p = 0.042; I2 = 81.32%).

#### Propofol vs Thiopental

We identified 3 studies comparing the effect of PROP administration (n = 63) to THIO (n = 57) on seizure duration^28^,^30^,^31^. There was no significance difference between the two groups (SMD = 0.433, 95% CI −0.367; 1.233, p = 0.289, I2 = 76.65%).

The associated funnel plot was reasonably symmetrical, although the limited number of studies does not allow the exclusion of publication bias (Appendix). The p value of the Egger’s regression intercept was >0.05.

After removing one study responsible for heterogeneity^31^, THIO was found to be significantly associated with longer seizure duration (SMD = 0.858, 95% CI 0.428–1.288, p < 0.001, I2 = 0.0%).

### Acceptability: dropouts’ rates

**Figure 5:**
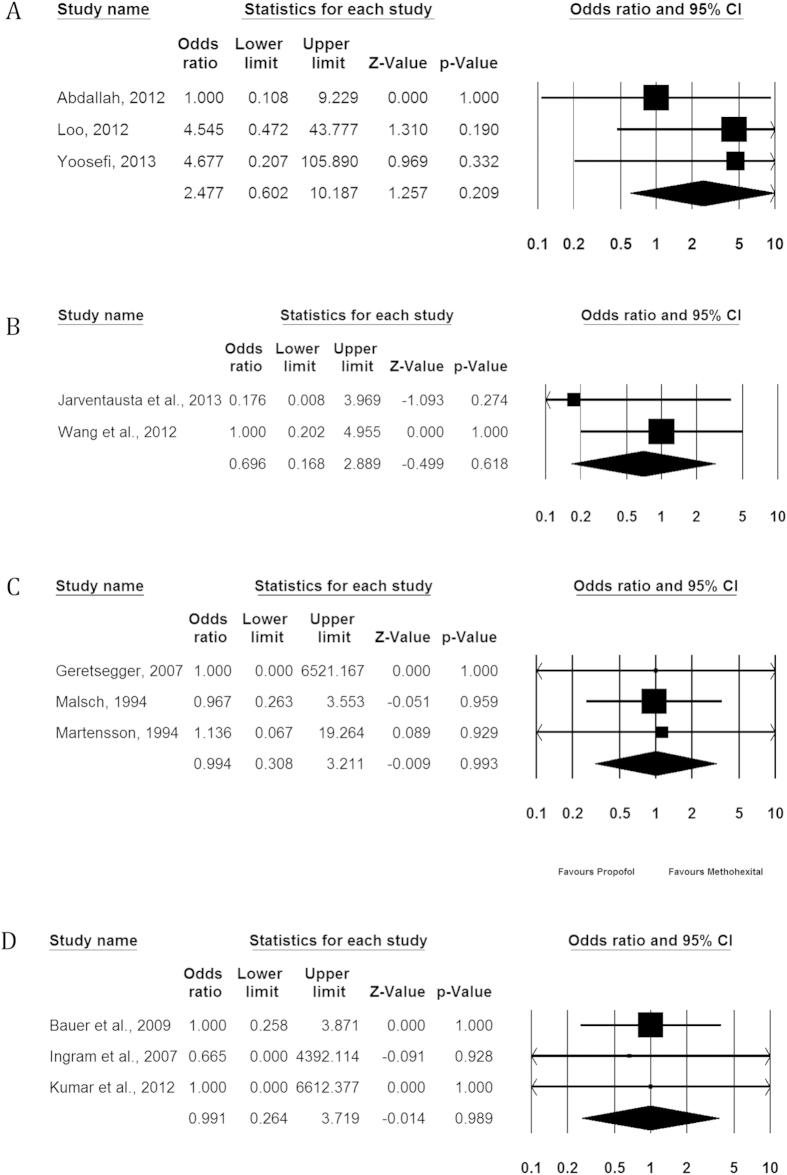
Comparative acceptability of the anesthetic agents, proxy by dropout rates. (**A**) Ketamine vs Thiopental. (**B**) Ketamine vs Propofol. (**C**) Propofol vs Methohexital. (**D**) Propofol vs Thiopental.

The data for dropout rates was available in 13 studies. (Table 3, Fig. 5).

#### Ketamine vs Thiopental

We identified 3 studies comparing the effect of KET administration (n = 52) to THIO (n = 49) on dropout^10^,^12^,^25^. There was no significant difference between the two groups (OR = 2.477, 95% CI 0.602; 10.187, p = 0.209 I2 = 0.0%).

The associated funnel plot was reasonably symmetrical, although the limited number of studies does not allow the exclusion of publication bias (Appendix). The p value of the Egger’s regression intercept was >0.05.

After removing a high risk of bias study^12^, the OR remained non significant (OR = 2.102, 95% CI 0.430–10.268, p = 0.359, I2 = 0.0%).

#### Ketamine vs Propofol

We identified 2 studies comparing the effect of KET administration (n = 32) to PROP (n = 32) on dropout rate^9^,^11^. There was no significant difference between the two groups (OR = 0.696, 95% CI 0.168; 2.889, p = 0.618, I2 = 0.0%).

#### Propofol vs Methohexital

We identified 3 studies comparing the effect of MET administration (n = 86) to PROP (n = 78) on dropout rate^29^,^32^,^33^. There was no significant difference between the two groups (OR = 0.994, 95% CI 0.308; 3.211, p = 0.993, I2 = 0.0%).

The associated funnel plot was reasonably symmetrical, although the limited number of studies does not allow the exclusion of publication bias (Appendix). The p value of the Egger’s regression intercept was >0.05, and the asymmetry is considered to be statistically non-significant.

#### Propofol vs Thiopental

We identified 3 studies comparing the effect of THIO administration (n = 57) to PROP (n = 63) on dropout rate^28^,^30^,^31^. There was no significant difference between the two groups (OR = 0.991, 95% CI 0.264; 3.719, p = 0.989, I2 = 0.0%).

The associated funnel plot was reasonably symmetrical, although the limited number of studies does not allow the exclusion of publication bias (Appendix). The p value of the Egger’s regression intercept was >0.05.

### Tolerability: adverse events

Adverse events were reported in 9 trials^9^,^10^,^11^,^12^,^25^,^26^,^29^,^31^,^33^ (Table 4) (Table 5).

Two studies used KET administration with high doses (respectively 0.8 mg/kg^11^ and 1–2 mg/kg^12^ and reported respectively 5 cardiovascular events (severe hypertension, diastole blood pressure >100 mmHg) and 2 dropouts for high tension. KET 0.5 mg/kg in combination to THIO was found to be associated with two cases of hypomanic and manic shifts in bipolar patients^10^. S-KET 0.4 mg/kg, an enantiomer of KET, associated with PROP, was found to be associated with one serious adverse events (hypertension+agitation) and with increased restlessness and disorientation^9^. Two studies suggested that THIO and METH were respectively associated with higher blood pressure increase compared to PROP^29^,^31^. In one study, both METH and PROP were associated with one delirious reaction^33^. Patients receiving ETO had more frequent adverse events (nausea, vomiting and allergy) than patients being administered THIO^26^. There was no reported data for MID.

### Tolerability: cognitive function

Cognitive function was assessed in 7 RCTs^10^,^12^,^27^,^28^,^30^,^31^,^33^. Two trials suggested that KET may be associated with moderate cognitive recovery after ECT compared to THIO. The latter was found to be associated with significant improvement in cognitive outcome compared to PROP^12^,^28^. (Table 5).

### Network meta-analyses to enable indirect comparisons

**Figure 6:**
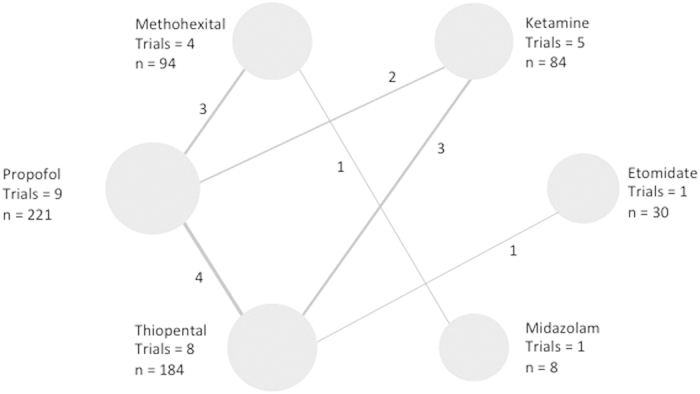
Network of induction agent comparisons in electroconvulsive therapy: Depression scores. Circle size reflects number of participants and the line width of the lines reflects the number of direct comparisons. No connecting line between 2 treatments indicates that there was no direct comparison.

**Figure 7:**
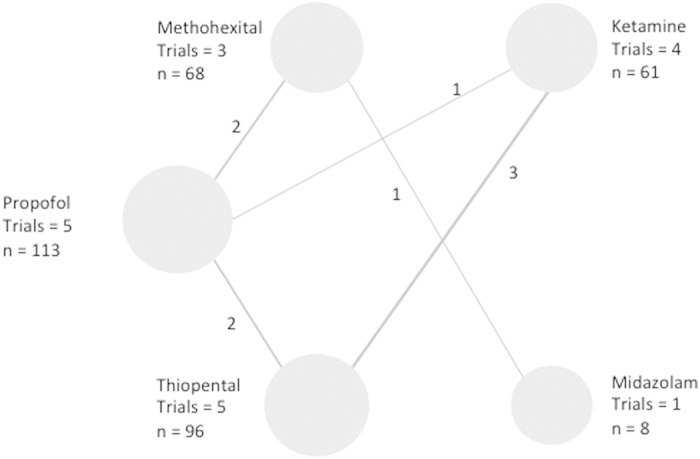
Network of induction agent comparisons in electroconvulsive therapy: Response rates. Circle size reflects number of participants and the line width of the lines reflects the number of direct comparisons. No connecting line between 2 treatments indicates that there was no direct comparison.

**Figure 8:**
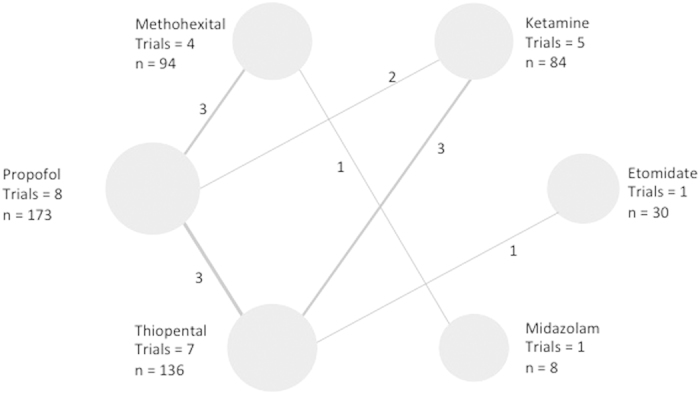
Network of induction agent comparisons in electroconvulsive therapy: Seizure durations. Circle size reflects number of participants and the line width of the lines reflects the number of direct comparisons. No connecting line between 2 treatments indicates that there was no direct comparison.

**Figure 9:**
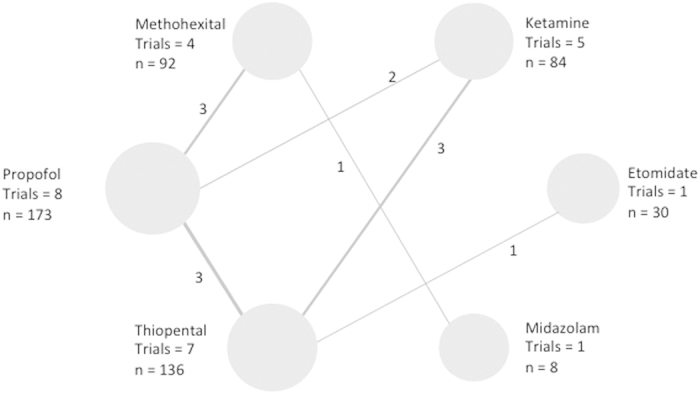
Network of induction agent comparisons in electroconvulsive therapy: dropout rates. Circle size reflects number of participants and the line width of the lines reflects the number of direct comparisons. No connecting line between 2 treatments indicates that there was no direct comparison.

(Figures 6, 7, 8, 9 and Table 6). Figures 6, 7, 8, 9 show the network geometry of eligible comparisons for the multiple-treatment meta-analysis, and Table 6 summarizes the efficacy (depression scores, responder rate and seizure duration) and acceptability (dropout rate) of the 6 anesthetic agents. The 6 anesthetic agents were not significantly different in term of efficacy on the depressive symptomatology, responder and dropout rate. Concerning seizure duration, METH was superior to PROP.

## Discussion

The aim of this Bayesian framework systematic review and meta-analysis was to compare the efficacy, tolerability and acceptability of 6 anesthetic agents in pre-ECT anesthesia induction in patients with major depression. Overall, 14 RCTs (621 patients) were included.

Our findings may be summarized as follows:

- Efficacy data: overall, no difference was found in baseline and post-treatment depression scores between the 6 anesthetic agents (all p > 0.05). However, after excluding trials responsible for heterogeneity, depression scores at 6 ECTs in patients who were administered METH were found to be significantly lower than those who received PROP (p = 0.001). Those who received PROP were lower than the one’s receiving THIO (p = 0.002). It was not possible to highlight the superiority of any anesthetic agent in regard of responder rate at the end of treatment, probably due to the lack of power and available data. METH was found to be associated with longer seizure duration compared to PROP. No other significant differences were found in regard of seizure duration.
- acceptability data: overall, no difference was found between the dropout rates of patients receiving KET, METH, PROP and THIO. There was no sufficient data for ETO and MID to conclude for these agents.
- tolerability data: The qualitative analysis of adverse events suggests that PROP appear as the safest agent in regard of blood pressure increase, compared to KET, THIO and METH. KET was specifically found to be associated with serious cardio-vascular adverse events, mostly hypertension, when administered alone in doses >=0.8 mg/kg. There was little evidence for better cognitive recovery after ECT with respectively KET compared to THIO and THIO compared to PROP.

Our major finding is that KET administration cannot be recommended to date as an anesthetic of choice for ECT anesthesia induction for depression, alone or in combination, due to the lack of efficacy data to date. Our results did not confirm the results from a non-randomized open-label trial that suggested KET administration being associated with lower post-treatment depression scores and earlier improvement^35^.

Given the serious cardio-vascular adverse events described in the included studies, KET doses >=0.8 mg/kg do not seem to be recommended. Further RCTs are needed to definitely determine if middle-doses KET (0.5 mg/kg) in adjunction may be useful or not. When comparing effectiveness data of trials co-administering KET with THIO^10^,^25^ and those which did not^11^,^12^, the result clearly suggest that KET effectiveness is improved when the doses rise and when KET is administered alone. As only one trial evaluated KET-PROP combination, with 0.4 mg/kg S-KET, with no significant results, it is not possible to conclude on the effectiveness of KET-PROP combination. A non-randomized controlled study recently suggested that KET-PROP administration may be associated with better antidepressive efficacy and less agitation and increased blood pressure if co-administered with dexmedetomidine^36^. This combination deserves further explorations.

Only one RCT^12^ comparing KET to THIO confirmed the putative pro-cognitive characteristics of KET that were previously suggested^37^,^38^. However KET was administered alone with high doses in this trial (>1 mg/kg), and a large number of cardiovascular events were described (5/17 = 29.4%).

Given the good effectiveness of KET administration in non-ECT patients 2, it may be administered apart from ECT sessions in patients with depression, in order to limit adverse events.

Efficacy data suggests that METH could be superior to PROP in post-treatment depression score. However, this significant result was found in only 2 RCTs, after removing one RCT for heterogeneity, and was not replicated in responder rate. Moreover, the number of patients was very limited. This result deserves further replication. This potential superior efficacy may be explained by longer seizure duration in patients being administered METH compared to PROP, as suggested by our results. After the studies responsible for heterogeneity were removed from the analysis, PROP was also found to be superior to THIO. However this result was found only in depression scores, as the data for responder rates was insufficient to conclude. THIO was associated with a superior seizure duration compared to PROP. The seizure duration may therefore not explain the superiority of PROP compared to THIO. THIO is a barbiturate anesthetic agent. In addition to this GABAergic effect, barbiturates have also been shown to block the signaling through the AMPA subtype of glutamate receptor^39^. It may be suggested that the GABA activity of THIO may explain lower effectiveness in depression scores for this anesthetic agent^40^,^41^. As only 2 RCTs were included after removing studies responsible for heterogeneity, further studies are also warranted to confirm the superiority of the effectiveness of PROP administration compared to THIO.

PROP was found to be associated with shorter seizure duration when compared to METH, but not with other anesthetic agents, which is consistent with the results of a retrospective study, which found that there was no difference between PROP, ETO and THIO in regard of seizure duration, cognitive recovery and cardiovascular events^2^.

One of the included studies suggested that THIO may be associated with better post-ECT cognition recovery^28^, compared to PROP. This was also suggested in a recent retrospective study^42^. It has also been recently suggested that ETO and KET may be associated with better seizure duration compared to PROP and THIO in retrospective data^43^. Nevertheless, data were not sufficient enough to conclude in our analysis.

Two manic switches in bipolar patients being administered KET were described in one study^10^. As manic switches may also be a side-effect of ECT treatment^44^, future studies should determine if KET administration may be associated with higher risk of manic switch in ECT-treated bipolar patients.

MID and ETO were respectively assessed in one trial only^26^,^27^. Data are not sufficient to conclude to the benefit/risk ratio of these anesthetic agents.

Limits. Our results should be interpreted with caution. The heterogeneity between studies’ designs limited our analyses (variable administered doses of anesthetic agents, MDD vs BD depression, ECT lead placement, number of ECT before assessment, depression scale used). The limited number of included patients was probably due to the difficulty to carry out ECT protocols in large samples. ECT is indicated mostly in pharmaceutical resistant depression, in consenting patients with no somatic contra-indication, and requires one anesthetist and one psychiatrist for each ECT session. Assessing efficacy by the responder rate is considered as the gold standard. However, this data was only available for 8 studies. As a result, we could not find a significant superiority of one anesthetic agent regarding the responder rate as primary outcome. As most of the studies were lacking this data, remitter’s rates were not analyzed in the present study. Only one study clearly mentioned that the 32 included patients received a diagnosis of resistant depression^9^. However, most of the other studies mentioned that the included patients were previously treated by antidepressants and/or mood stabilizers, with persistent high depressive scores before the ECT treatment. However, the mean number of previous antidepressants treatment was not mentioned in most of the studies. It was therefore not possible to carry on subgroup analyses comparing resistant and non-resistant depression. Seven studies included 53 patients with bipolar depression (Table 1), but these patients were mixed with patients with unipolar depression. One study also included patients with a diagnosis of depression with psychotic features^31^. As the analyses were carried out on global results, it was not possible to discriminate bipolar from unipolar depression. Some of these scales include anxiety and sleep items. Analyzing only depressive core symptoms on each scale would have been worth of interest. However, individual data was not available in the present work. Two studies used only the BDI scale to assess depressive scores^26^,^31^ (Table 1). BDI is an autoquestionnaire. Comparing hetero and autoquestionnaire may be questionable. However, the scores on depression scales were not used as a responder criterion in these two studies (Table 3). Removing these studies from the analyses did not change our major findings (Fig. 2). The time point after 6 ECT sessions was chosen when available in the papers. However, this data was available in 2 studies only (Table 3). The depression scores at the end of treatment were found in 7 studies and included in the analyses. This may also be responsible for heterogeneity.

Altogether, these limits suggest that future studies should pay attention on the definition and characteristics of the depression of the included patients (especially the number of previous administered antidepressants). Both hetero and autoevaluation of depression should be included, and depression core symptoms may be analyzed separately.

### Strengths

Only double-blind RCTs were included in this Bayesian framework meta-analysis. Thirteen out of 14 RCTs were classified as good quality level RCTs (the last one was deleted in sensitivity analyses but this did not change our results). All these RCTs had a dropout rate <20%, masked outcome assessment by validated depression scales, baseline equivalent characteristics, clear inclusion/exclusion criteria and concealed allocation. Most of the quality lack was due to insufficient description of randomization methodology.

### Conclusion

Current data is insufficient at the moment to recommend one specific anesthetic agent in the induction of anesthesia for ECT in major depressive episode. Larger well-designed randomized studies are needed to determine which intravenous anesthetic medication leads to the greatest improvement in depression scores with minimal adverse events. Anesthetic agents should be chosen on the basis of adverse events profile, emergence and how these medications affect seizure duration.

## Additional Information

**How to cite this article**: Fond, G. *et al.* A Bayesian framework systematic review and meta-analysis of anesthetic agents effectiveness/tolerability profile in electroconvulsive therapy for major depression. *Sci. Rep.* **6**, 19847; doi: 10.1038/srep19847 (2016).

## Figures and Tables

**Table 1:** Study characteristics.

Year	Author	Design	Country	Population	Diagnosis	ECT lead placement	N MDD	N BP	Depression Scale
2012	Abdallah	DB-RCT	USA	MDD+BD	DSMIV	unilateral/bilateral	10	6	25-HDRS
2012	Abdohalli	DB-RCT	Iran	MDD	DSMIV	bilateral	60	0	BDI
1995	Auriacombe	DB-RCT	France	MDD	DSM-III-R	bilateral	16	0	MADRS
2009	Bauer	DB-RCT	Denmark	MDD+BD	CIM10	unilateral/bilateral	47	15	17-HDRS
2007	Geretsegger	DB-RCT	Austria	MDD+BD	DSM-III-R	unilateral	37	13	HDRS
2007	Ingram	DB-RCT	Australia	MDD+BD	DSMIV	unilateral/bilateral	24	6	17-HDRS
2013	Jarventausta	DB-RCT	Finland	resistant MDD*	DSMIV	unilateral/bilateral	32	0	MADRS
2012	Kumar	DB-RCT	India	MDD+BD+SZ +2somatoform +2transient psychosis	CIM10	bilateral	14	6	BDI
2012	Loo	DB-RCT	Australia	MDD+BD	DSMIV	unilateral	37	9	MADRS
1994	Malsch	DB-RCT	USA	MDD	DSM-III-R	unilateral/bilateral	58	0	HDRS
1994	Martensson	DB-RCT	Sweden	MDD+BD	DSM III	unilateral	37	10	MADRS
2013	Purtuloglu	DB-RCT	Turkey	unclear	DSMIV	bilateral	96	0	HDRS
2012	Wang	DB-RCT	China	MDD	DSMIV	bilateral	48	0	17-HDRS
2013	Yoosefi	DB-RCT	Iran	MDD	DSMIV	bilateral	29	0	21-HDRS

DB-RCT: double-blind randomized controlled trial. MDD major depressive disorder. BD Bipolar Disorder. *including 10 patients with depression with psychotic features.

**Table 2:** Summary of study methodology using the Cochrane Risk of Bias Tool and the classification scheme requirements for therapeutic questions.

Author	The classification scheme requirements for therapeutic questions								The Cochrane Risk of Bias Tool				
	R	MOA	BE	CA	PO	EID	D	Level	SG	AC	B	COD	Risk
Abdohalli 2012	Y	Y	Y	Y	Y	Y	Y	**Class I**	Y	Y	Y	Y	**Low risk of bias**
Abdallah 2012	Y	Y	Y	Y	Y	Y	Y	**Class I**	Y	Y	Y	Y	**Low risk of bias**
Auriacombe 1995	U	Y	Y	Y	Y	Y	Y	**Class II**	U	Y	Y	Y	**Unclear risk of bias**
Bauer 2009	Y	Y	Y	Y	Y	Y	Y	**Class I**	Y	Y	Y	Y	**Low risk of bias**
Geretsegger 2007	U	Y	Y	Y	Y	Y	Y	**Class II**	U	Y	Y	Y	**Unclear risk of bias**
Ingram 2007	Y	Y	Y	Y	Y	Y	Y	**Class I**	Y	Y	Y	Y	**Low risk of bias**
Jarventausta 2013	U	Y	Y	Y	Y	Y	Y	**Class II**	U	Y	Y	Y	**Unclear risk of bias**
Kumar 2012	Y	Y	Y	Y	Y	Y	Y	**Class I**	U	Y	Y	Y	**Unclear risk of bias**
Loo 2012	Y	Y	Y	Y	Y	Y	Y	**Class I**	Y	Y	Y	Y	**Low risk of bias**
Malsch 1994	U	Y	Y	Y	Y	Y	Y	**Class II**	U	Y	Y	Y	**Unclear risk of bias**
Martensson 1994	Y	Y	Y	Y	Y	Y	Y	**Class I**	Y	Y	Y	Y	**Low risk of bias**
Purtuloglu 2013	U	U	U	U	U	U	U	**Class IV**	U	U	Y	U	**High risk of bias**
Wang 2012	Y	Y	Y	Y	Y	Y	Y	**Class I**	Y	Y	Y	Y	**Low risk of bias**
Yoosefi 2013	Y	Y	Y	Y	Y	Y	Y	**Class I**	Y	Y	Y	Y	**Low risk of bias**

Y: Yes; N: No, U: unclear.

R: randomization; MOA: Masked or objective Outcome Assessment; BE: Baseline Equivalent characteristics or appropriate adjustment; CA: Concealed Allocation; PO: Primary Outcome clearly defined; EID: Exclusion/Inclusion clearly Defined; D: Dropouts < 20%.

SG: Sequence Generation; AC: Allocation Concealment; B: Blinding; COD: Complete Outcome data.

**Table 3:** Reported rates of responders and dropouts.

Author	Response definition	Timepoint to response assessment	Group 1 Anaesthetic type, mean dose (mg/kg)	% responders group 1	% dropouts group 1	Group 2 Anaesthetic type, mean dose (mg/kg)	% responders group 2	% dropouts group 2
Abdallah	>=50% HDRS	6 ECT	KET 0.5 mg/kg + 3.5 mg/kg THIO	0 (0/9)	0.22 (2/9)	THIO 3.5 mg/kg	0.13 (1/9)	0.22 (2/9)
Abdohalli	none	—	ETO 0.2 mg/kg	—	0 (0/30)	THIO 3 mg/kg	—	0 (0/30)
Auriacombe	>55% MADRS	End of treatment	METH 1 mg/kg	0.75 (6/8)	0.11 (1/6)	MID 0.1 mg/kg	0.875 (7/8)	0.11 (1/8)
Bauer	>=50% HDRS	End of treatment	PROP 1.5 mg/kg	0.16 (5/31)	0.16 (5/31)	THIO 3 mg/kg	0.195 (6/31)	0.16 (5/31)
Geretsegger	>=50% HDRS	End of treatment	METH 1.43 mg/kg	0.76 (19/25)	0 (0/25)	PROP 1.72 mg/kg	0.6 (15/25)	0 (0/25)
Ingram	>=60% HDRS	End of treatment	PROP 1–2 mg/kg	0.64 (7/11)	0 (0/18)	THIO 2–4 mg/kg	0.28 (8/18)	0 (0/12)
Jarventausta	>=50% HDRS	End of treatment	S-KET 0.4 mg/kg + PROP	0.929 (13/16)	0 (0/16)	PROP 0.5mg/kg +10mg/10sec	0.846 (11/16)	0.13 (2/16)
Kumar	none	—	THIO 3mg/kg	—	0 (0/14)	PROP 1.5 mg/kg	—	0 (0/14)
Loo	>=50% MADRS	6 ECT	KET 0.5 mg/kg + THIO	0.474 (9/19)	0.15 (4/26)	THIO 3–5 mg/kg	0.542 (13/24)	0.04 (1/26)
Malsch	final HDRS<=18	End of treatment	METH 0.75–1.5 mg/kg	0.886 (31/35)	0.17 (6/35)	PROP 1–2.5 mg/kg	0.967 (29/30)	0.17 (5/30)
Martensson	none	—	METH 1.05 mg/kg	—	0.038 (1/26)	PROP 1.32 mg/kg	—	0.043 (1/23)
Purtuloglu	none	—	PROP*	—	—	THIO*	—	—
Wang	none	—	KET 0.8 mg/kg, once	—	0.25 (4/16)	PROP 1.5 mg/kg	—	0.25 (4/16)
Yoosefi	>=60% HDRS	End of treatment	KET 1–2 mg/kg	0 (0/17)	0.12 (2/17)	THIO 2–3 mg/kg	0 (0/14)	0 (0/14)

ECT: electro-convulsive therapy. ETO etomidate, METH methohexital, KET ketamine, PROP propofol, THIO thiopental. HDRS Hamilton Depression Rating Scale. MADRS Montgomery-Asberg Depression Rating Scale.

*doses were not reported and authors could not be contacted.

**Table 4:** Reported adverse events (adverse events were not reported in the 5 other studies).

Author	Group 1 Anaesthetic type, mean dose (mg/kg)	Group 2 Anaesthetic type, mean dose (mg/kg)	Reported adverse events
Abdallah	KET 0,5 mg/kg + 3.5 mg/kg THIO	THIO 3.5 mg/kg	No major adverse effects were observed in this cohort during the 2 weeks of ECT treatment. Minimal transient side effects reported by both groups included nausea, headaches, disorientation, and muscle pain.
Abdohalli	ETO 0.2 mg/kg	THIO 3 mg/kg	Nausea and vomiting (5 ETO vs 3 THIO), myoclonus (3 ETO vs 0 THIO), allergy (3 ETO vs 2 THIO)
Geretsegger	METH 1.43 mg/kg	PROP 1.72 mg/kg	The increase in blood pressure was much more moderate in PROP group compared to METH group.
Loo	KET (0.5 mg/kg) + THIO 3–5 mg/kg	THIO 3–5 mg/kg	No psychomimetic effects were reported after KET administration and KET did not significantly increase post-treatment agitation or confusion. Of the nine BD participants, one became hypomanic and one developed rapid cycling manic symptoms. Both were in the KET group and were on lithium at therapeutic serum levels during the course of ECT.
Jarventausta	S-KET (0,4 mg/kg) + PROP	PROP 0.5 mg/kg +10 mg/10sec	The post treatment disorientation and restlessness seemed to be more common in the S-KET group. In the S-KET group, there was 1 serious adverse event (agitation, hyperventilation, sense of fear, raise of blood pressure, and heart rate).
Kumar	THIO 3 mg/kg	PROP 1.5 mg/kg	The percentage increase in each of the variables following the procedure (blood pressure) was significantly greater in the group THIO as compared to the group PROP.
Martensson	METH 1.05 mg/kg	PROP 1.32 mg/kg	The 2 dropouts were due to delirious reaction (1 PROP and 1 METH)
Wang	KET 0.8 mg/kg, once	PROP 1.5 mg/kg	There were 5 cardiovascular events (severe hypertension, diastole blood pressure >100 mmHg) during ECT in ketamine group requiring 25 mg urapidil (intravenous). Headache occurred in 6 patients (50%), nausea in 3 (25%) patients, brief delirium (within 2hours) in 2 patients, prolonged delirium (>2 h) in one patient and sense of fear upon awakening from anaesthesia in 3 (25%) patients. Only hypertension was only significantly enhanced in KET group compared to controls (p = 0.037).
Yoosefi	KET 1–2 mg/kg	THIO 2–3 mg/kg	2 dropouts due to high blood pression in KET group, one in THIO group.

ECT: electro-convulsive therapy. ETO etomidate, METH methohexital, KET ketamine, PROP propofol, THIO thiopental.

**Table 5:** Cognitive function outcomes.

Author	Cognitive evaluation	Findings
Auriacombe	MMSE	There were no statistically significant differences in the evolution of memory performance between METH and MID groups throughout the ECT course.
Bauer	MMSE	MMSE score was significantly lower in the PROP group compared to the THIO group.
Ingram	Verbal memory, visual memory, language, speed and attention testes	No significant differences between PROP and THIO groups
Kumar	MMSE	No significant differences between PROP and THIO groups
Loo	Medical College of Georgia Complex Figure (CFT); Hopkins Verbal Learning Test (HVLT); Controlled Oral Word Association Test (COWAT); Symbol Digit Modalities Test (SDMT); Woodcock Johnson Cross-Out Test; Autobiographical Memory Interview—short form (AMI-SF).	No significant differences between KET+THIO and THIO groups
Martensson	MMSE WMS logical prose Rey.Osterrieth Corsi blocks Knox cubes Buschke’s cued call Claeson-Dahl learning Verbal fluency	No significant differences between PROP and METH groups.
Yoosefi	MMSE	Superior improvement in KET group compared to THIO group

MMSE Mini Mental Status Examination. KET ketamine METH methohexital MID midazolam PROP propofol THIO thiopental.

**Table 6:** Comparison of anesthesia induction agent effects in electroconvulsive therapy (ECT).

Induction agents	Depression mean score difference (95% CI)	Response Odds Ratio (95% CI)	Drop out Odds Ratio (95% CI)	Seizure duration mean difference (95% CI)
Ketamine vs Etomidate	0.2 (−8.1; 8.3)	—	0.2 (9.4 ^e−21^; 1.3^e+14^)	−1.6 (−24; 21)
Methohexital vs Etomidate	3.5 (−5.7; 13)	—	0.2 (1.1 ^e−20^; 1.7^e+14^)	6.5 (−19; 33)
Propofol vs Etomidate	0.5 (−7.9; 8.6)	—	0.3 (9.3 ^e−21^; 2.1^e+14^)	−11 (−33; 13)
Thiopenthal vs Etomidate	0.6 (−7.0; 8.0)	—	0.1 (5.0 ^e−21^; 7.4^e+13^)	−5.8 (−25; 14)
Midazolam vs Etomidate	3.3 (−8.2; 15)	—	0.2 (3.9 ^e−21^; 1.3^e+14^)	5.5 (−27; 40)
Etomidate vs Ketamine	−0.2 (−8.3; 8.1)	—	4.6 (7.7 ^e−15^; 1.1^e+20^)	1.6 (−21; 24)
Methohexital vs Ketamine	3.3 (−2.4; 9.1)	1.2 (0.2; 8.4)	1.1 (0.1; 17)	8.2 (−8.1; 25)
Propofol vs Ketamine	0.3 (−3.5; 4.0)	1.2 (0.3; 5.3)	1.1 (0.2; 7)	−9.6 (−20; 2.2)
Thiopenthal vs Ketamine	0.4 (−3.1; 3.9)	1.2 (0.3; 4.5)	0.5 (0.1; 2.2)	−4.2 (−15; 6.1)
Midazolam vs Ketamine	3.2 (−6.4; 0.1)	3.5 (0.1; 2.4^e+02^)	0.8 (0.1; 1.0^e+02^)	7 (−19; 35)
Etomidate vs Methohexital	−0.2 (−8.3; 8.1)	−	4.6 (5.9 ^e−15^; 9.3^e+19^)	−6.5 (−33; 19)
Ketamine vs Methohexital	3.3 (−2.4; 9.1)	0.8 (0.1; 6.5)	0.9 (0.1; 10)	−8.2 (−25; 8.1)
Propofol vs Methohexital	0.3 (−3.5; 4.0)	1.0 (0.3; 4.4)	1.0 (0.1; 6.6)	−**18 (**−**30;**−**5.2)**
Thiopenthal vs Methohexital	0.4 (−3.1; 3.9)	1.0 (0.2; 7)	0.5 (0.0; 4.9)	−12 (−30; 3.0)
Midazolam vs Methohexital	3.1 (−6.4; 13)	2.8 (0.1; 1.2^e+02^)	0.7 (0.0; 39)	−1.1 (−22; 20)
Etomidate vs Propofol	−3.5 (−13; 5.7)	−	3.9 (4.9 ^e−15^; 1.1^e+20^)	11 (−13; 33)
Ketamine vs Propofol	−3.3 (−9.1; 2.4)	0.8 (0.2; 3.6)	0.9 (0.1; 4.5)	9.6 (−2.2; 20)
Methohexital vs Propofol	−3.1 (−7.5; 1.2)	1.0 (0.2; 4.0)	1.0 (0.2; 6.9)	**18 (5.2; 30)**
Thiopenthal vs Propofol	−2.9 (−8.4; 2.3)	1.0 (0.3; 3.5)	0.5 (0.1; 2.2)	5.4 (−6.4; 15)
Midazolam vs Propofol	−0.1 (−7.7; 7.2)	2.9 (0.1; 1.7^e+02^)	0.7 (0.0; 61)	17 (−7.3; 41)
Etomidate vs Thiopenthal	−0.5 (−8.6; 7.9)	—	8.3 (1.4 ^e−14^; 2.0^e+20^)	5.8 (−14; 25)
Ketamine vs Thiopenthal	−0.3 (−4.0; 3.5)	0.8 (0.2; 3)	2.0 (0.5; 10)	4.2 (−6.1; 15)
Methohexital vs Thiopenthal	3.1 (−1.2; 7.5)	1.0 (0.1; 6)	2.2 (0.2; 39)	12 (−3.0; 30)
Propofol vs Thiopenthal	0.1 (−3.1; 3.3)	1.0 (0.3; 3.4)	2.1 (0.5; 16)	−5.4 (−15; 6.4)
Midazolam vs Thiopenthal	2.9 (−5.8; 12)	2.9 (0.8; 1.9^e+02^)	1.6 (0.1; 2.1^e+02^)	11 (−14; 39)
Etomidate vs Midazolam	−0.6 (−8.0; 7.0)	—	5.9 (7.8 ^e−15^; 2.5^e+20^)	−5.5 (−40; 27)
Ketamine vs Midazolam	−0.4 (−3.9; 3.1)	0.3 (0.0; 12)	1.3 (0.0; 1.9^e+02^)	−7 (−35; 19)
Methohexital vs Midazolam	2.9 (−2.3; 8.4)	0.4 (0.1; 8)	1.5 (0.3; 1.1^e+02^)	1.1 (−20; 22)
Propofol vs Midazolam	−0.1 (−3.3; 3.1)	0.3 (0.0; 10)	1.4 (0.02; 1.7^e+02^)	−17 (−41; 7.3)
Thiopenthal vs Midazolam	2.8 (−6.4; 12.)	0.3 (0.0; 14)	0.6 (0.0; 89)	−11 (−39; 14)

Significant associations (p < 0.05) are in bold.
